# Dedicated Axillary MRI-Based Radiomics Analysis for the Prediction of Axillary Lymph Node Metastasis in Breast Cancer

**DOI:** 10.3390/cancers13040757

**Published:** 2021-02-12

**Authors:** Sanaz Samiei, Renée W. Y. Granzier, Abdalla Ibrahim, Sergey Primakov, Marc B. I. Lobbes, Regina G. H. Beets-Tan, Thiemo J. A. van Nijnatten, Sanne M. E. Engelen, Henry C. Woodruff, Marjolein L. Smidt

**Affiliations:** 1 Department of Surgery, Maastricht University Medical Center+, P.O. Box 5800, 6202 AZ Maastricht, The Netherlands; snz.samiei@gmail.com (S.S.); sanne.engelen@mumc.nl (S.M.E.E.); m.smidt@mumc.nl (M.L.S.); 2 Department of Radiology and Nuclear Medicine, Maastricht University Medical Center+, P.O. Box 5800, 6202 AZ Maastricht, The Netherlands; a.ibrahim@maastrichtuniversity.nl (A.I.); s.primakov@maastrichtunversity.nl (S.P.); marc.lobbes@mumc.nl (M.B.I.L.); thiemo.nijnatten@mumc.nl (T.J.A.v.N.); h.woodruff@maastrichtuniversity.nl (H.C.W.); 3 GROW-School for Oncology and Developmental Biology, Maastricht University, P.O. Box 616, 6200 MD Maastricht, The Netherlands; r.beetstan@nki.nl; 4 The D-Lab, Department of Precision Medicine, Maastricht University, Universiteitssingel 40, 6229 ER Maastricht, The Netherlands; 5 Division of Nuclear Medicine and Oncological Imaging, Department of Medical Physics, Hospital Center Universitaire de Liege, Rue de Gaillarmont 600, 4030 Liege, Belgium; 6 Department of Nuclear Medicine and Comprehensive Diagnostic Center Aachen (CDCA), University Hospital RWTH Aachen University, Pauwelsstrasse 30, 52074 Aachen, Germany; 7 Department of Medical Imaging, Zuyderland Medical Center, P.O. Box 5500, 6130 MB Sittard-Geleen, The Netherlands; 8 Department of Radiology, The Netherlands Cancer Institute, P.O. Box 90203, 1006 BE Amsterdam, The Netherlands

**Keywords:** dedicated axillary MRI, axillary lymph node metastasis, node-by-node matching, radiomics, predictive modeling

## Abstract

**Simple Summary:**

The presence of axillary lymph node metastases in breast cancer patients is an essential factor in axillary surgery and possible additional treatment. This study aimed to investigate the potential of dedicated axillary MRI-based radiomics analysis for the prediction of axillary lymph node metastases. Dedicated axillary MRI examinations provide a very specific and complete field of view of the axilla. Accurate preoperative prediction of axillary lymph node metastases in breast cancer patients using radiomics analysis can aid in clinical decision-making for the type of treatment.

**Abstract:**

Radiomics features may contribute to increased diagnostic performance of MRI in the prediction of axillary lymph node metastasis. The objective of the study was to predict preoperative axillary lymph node metastasis in breast cancer using clinical models and radiomics models based on T2-weighted (T2W) dedicated axillary MRI features with node-by-node analysis. From August 2012 until October 2014, all women who had undergone dedicated axillary 3.0T T2W MRI, followed by axillary surgery, were retrospectively identified, and available clinical data were collected. All axillary lymph nodes were manually delineated on the T2W MR images, and quantitative radiomics features were extracted from the delineated regions. Data were partitioned patient-wise to train 100 models using different splits for the training and validation cohorts to account for multiple lymph nodes per patient and class imbalance. Features were selected in the training cohorts using recursive feature elimination with repeated 5-fold cross-validation, followed by the development of random forest models. The performance of the models was assessed using the area under the curve (AUC). A total of 75 women (median age, 61 years; interquartile range, 51–68 years) with 511 axillary lymph nodes were included. On final pathology, 36 (7%) of the lymph nodes had metastasis. A total of 105 original radiomics features were extracted from the T2W MR images. Each cohort split resulted in a different number of lymph nodes in the training cohorts and a different set of selected features. Performance of the 100 clinical and radiomics models showed a wide range of AUC values between 0.41–0.74 and 0.48–0.89 in the training cohorts, respectively, and between 0.30–0.98 and 0.37–0.99 in the validation cohorts, respectively. With these results, it was not possible to obtain a final prediction model. Clinical characteristics and dedicated axillary MRI-based radiomics with node-by-node analysis did not contribute to the prediction of axillary lymph node metastasis in breast cancer based on data where variations in acquisition and reconstruction parameters were not addressed.

## 1. Introduction

In breast cancer patients, the axillary lymph node status provides essential prognostic information about the locoregional recurrence and overall survival rate [1,2,3,4]. The five-year survival rate decreases from 99% to 85% with the presence of lymph node metastasis in the axilla [5]. The presence of axillary lymph node metastasis determines the extent of the surgical treatment plan, the potential need for (neo)adjuvant systemic therapy, and the possible indication for postmastectomy radiation therapy with regard to immediate breast reconstruction [6,7].

In the preoperative setting, imaging for axillary lymph node assessment is recommended in the clinical workup of invasive breast cancer patients [6]. For the evaluation of tumor extent in the breast or following neoadjuvant treatment, breast magnetic resonance imaging (MRI) is often performed, which includes the axilla in the field of view [8]. However, when using dedicated breast coils, the field of view of the axillary region can be limited [9]. Therefore, dedicated MR coils for visualization and assessment of the axillary region have been investigated [10,11,12]. Dedicated unenhanced T2-weighted (T2W) axillary MRI showed good diagnostic performance based on node-by-node analysis but remained insufficient to accurately exclude axillary lymph node metastasis [12].

Although preoperative imaging may be performed to guide the axillary management of patients, no current imaging modality with optimal diagnostic performance can replace the surgical axillary staging procedure. In the era of artificial intelligence, current developments in radiology focus on the improvement of decision support systems to maximize the potential role of noninvasive imaging modalities. Radiomics, the application of machine learning to medical imaging, is a rapidly evolving field that enables high-throughput quantitative data extraction from standard medical images in an automated fashion and subsequent data analysis, possibly combined with patient and tumor characteristics, improving the accuracy of diagnostic, predictive, and prognostic models [13,14]. The evaluation of the usefulness of radiomics based on mammography, ultrasound, and breast MRI has been explored, showing potential in axillary lymph node metastasis prediction [15,16,17,18,19]. However, this research focused on the prediction of axillary lymph node metastasis from the delineated breast tumor as the region of interest (ROI), and not from the lymph nodes themselves.

Accurate preoperative prediction of axillary lymph node metastasis in breast cancer patients can assist in clinical decision-making regarding the type of treatment. Radiomics features extracted from axillary lymph nodes may contribute to increased diagnostic performance of MRI in the prediction of metastasis. To our knowledge, no previous study has reported on node-by-node matching of axillary lymph nodes with pathological findings in breast cancer patients in the field of radiomics. The purpose of this study was to predict preoperative axillary lymph node metastasis in breast cancer patients using clinical models and radiomics models based on unenhanced T2W dedicated axillary MRI features with node-by-node analysis.

## 2. Results

### 2.1. Patients Characteristics

A total of ninety women were considered for inclusion, of whom twelve were excluded due to treatment with neoadjuvant systemic therapy before axillary surgery and three with ductal carcinoma in situ only. Seventy-five patients (median age, 61 years; interquartile range, 51–68 years) with 511 axillary lymph nodes were included. Patient, tumor, and treatment characteristics are summarized in Table 1. The median number of axillary lymph nodes per patient was six, with a range of 1–18. Fourteen of the included patients were node-positive at final pathology, with a total of 36 axillary lymph nodes with macrometastases and 58 axillary lymph nodes without metastasis. The remaining 61 patients had 417 axillary lymph nodes without metastasis. The median number of voxels per ROI for all delineated axillary lymph nodes was 100 (interquartile range, 44–236) and 310 (interquartile range, 130–1676) for all delineated axillary lymph nodes with metastasis. The Spearman correlation between the number of voxels per ROI and the corresponding pathological outcome was 0.22.

### 2.2. Radiomics Feature Extraction and Model Development

**Figure 1:**
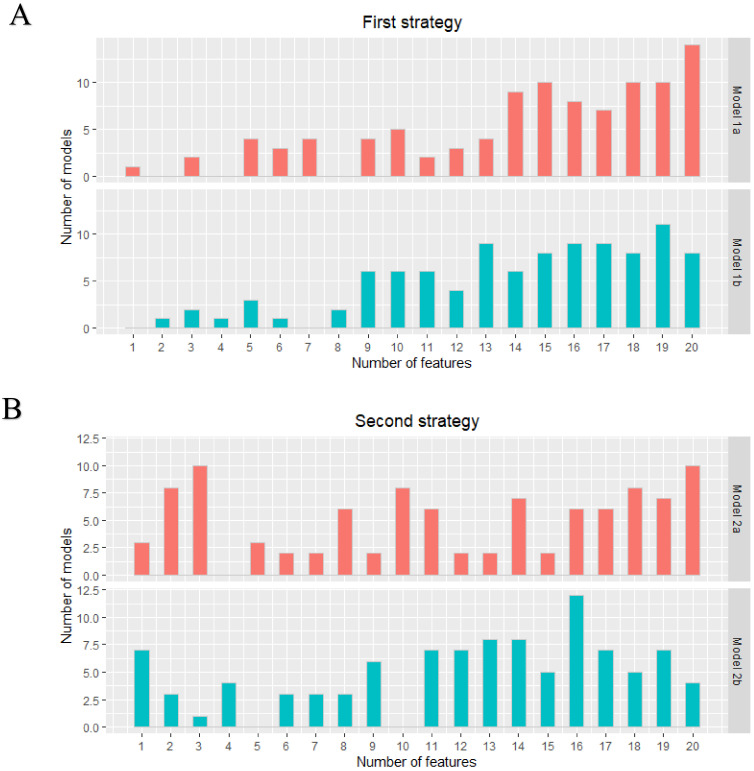
First (**A**) and second (**B**) strategy: distribution of the number of features in each developed model. The two different models in both strategies were all developed 100 times.

A total of 105 original radiomics features were extracted from the dedicated axillary T2W MR images. No near-zero variance features were detected. Pearson pairwise correlation removed 53 highly correlated features. The optimal subset of features was selected in the training cohort using recursive feature elimination with repeated 5-fold cross-validation with a maximum of 20 features. Figure 1 shows the distribution of the number of selected features from the 100 iterations for the two different strategies (lymph nodes from all patients versus only lymph nodes from node-positive patients as data points) for each model. Supplementary Material A includes a list of how often each feature was chosen in the 100 iterations for each model.

**Figure 2:**
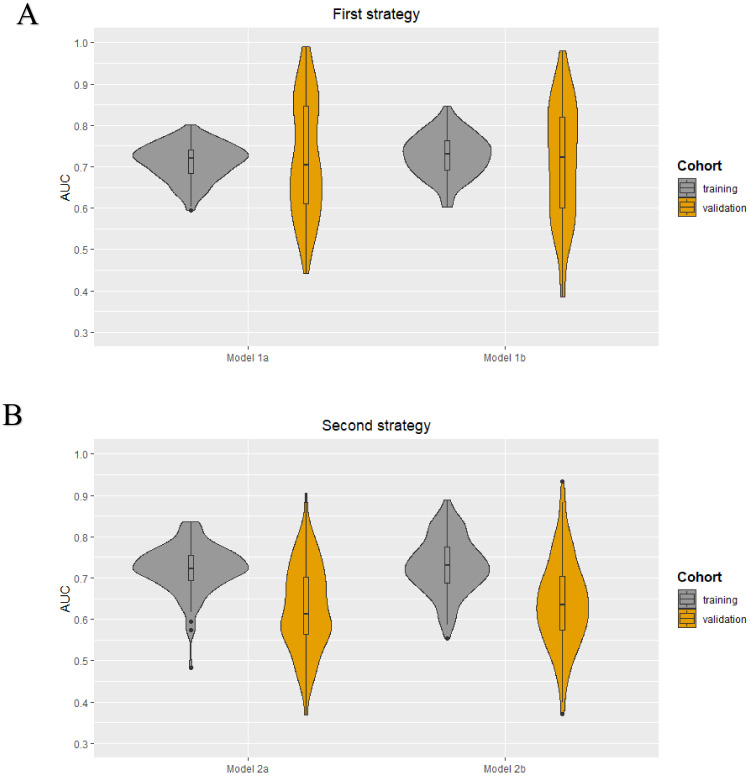
Violin plots for the radiomics models developed using the first (**A**) and second (**B**) strategy: AUC value distribuTable 100. iterations) for the four models (1a, 1b, 2a, and 2b) in both the training and validation cohort.

As each iteration resulted in a different set of selected features for each model in both strategies, it was not possible to obtain a final prediction model. The minimum and maximum area under the curve (AUC) values in the training cohorts were 0.59–0.80, 0.60–0.85, 0.48–0.84, and 0.55–0.89 for models 1a, 1b, 2a, and 2b, respectively. The median AUC values for all models in the training cohorts were between 0.72–0.73. All models showed a wider range of AUC values in the validation cohorts. The AUC value distribution for all models in the training and validation cohorts are presented in the violin plots in Figure 2. The minimum and maximum sensitivity in the training cohorts were 30–66%, 53–83%, 7–74%, and 48–82% for models 1a, 1b, 2a, and 2b, respectively. The median sensitivity for all models in the training cohorts was between 47–66%. All models showed lower median sensitivity in the validation cohorts. The minimum and maximum PPV in the training cohorts were 46–78%, 55–83%, 25–80%, and 52–90% for models 1a, 1b, 2a, and 2b, respectively. The median PPV for all models in the training cohorts were between 61–67%. All models showed a lower median PPV in the validation cohorts. The diagnostic performance parameters of the radiomics models (100 iterations) are shown in Table 2.

The additional feature selection step with the cut-off values >0.75, >0.80, and >0.90 resulted in 44, 35, and 8 original features, respectively, available for recursive feature elimination with repeated 5-fold cross-validation. These results showed no differences compared to the results found without this additional feature selection step. The violin plots of the models developed after adding the additional feature selection step can be found in Figures S1–S3.

### 2.3. Radiomics Subanalysis

After the exclusion of ROIs with less than 50 voxels, a total of 71 patients were included for analyses, with 371 axillary lymph nodes. Thirteen of these patients were node-positive, with a total of 31 axillary lymph nodes with metastasis and 34 axillary lymph nodes without metastases. The remaining 58 patients had 340 axillary lymph nodes without metastasis. Excluding small lymph nodes resulted in balanced training cohorts in models 1a and 2a, eliminating the need to perform random undersampling (models 1b and 2b). The minimum and maximum AUC values of the balanced models 1a and 2a in the training and validation cohorts of this subanalysis were 0.53–0.82 and 0.41–0.83, respectively. Violin plots with the distribution of the AUC values and the diagnostic performance parameters of the subanalysis are provided in Table S1 and Figure S4.

### 2.4. Clinical Model Development

**Figure 3:**
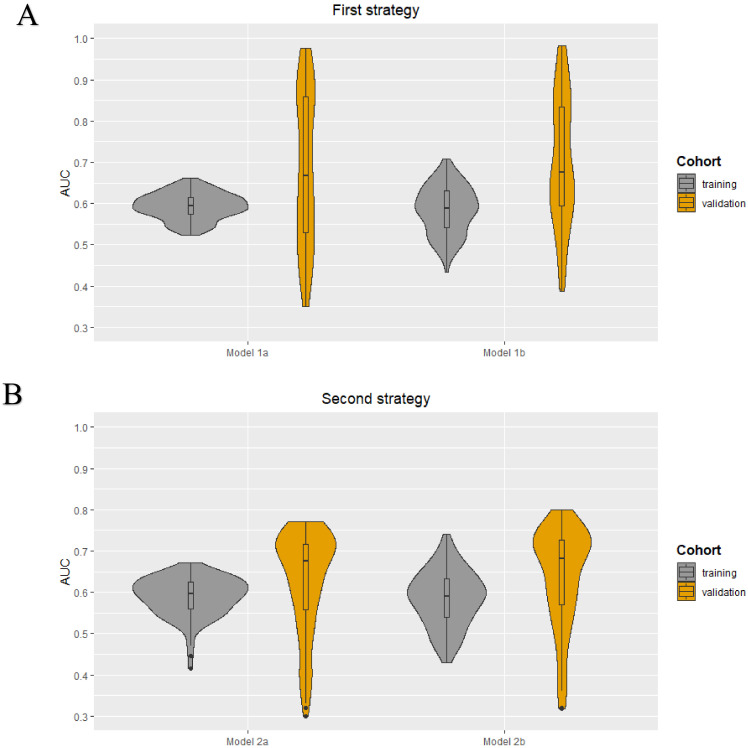
Violin plots for the clinical models developed using the first (**A**) and second (**B**) strategy: AUC value distributions (100 iterations) for the four models (1a, 1b, 2a, and 2b) in both the training and validation cohort.

The following clinical characteristics were available and selected for the development of the clinical models: patient age, clinical tumor size, clinical tumor stage, tumor histology, tumor grade, and receptor subtype (ER, PR, and HER2+). No highly correlated clinical characteristics were present. The minimum and maximum AUC values in the training cohorts were 0.52–0.66, 0.43–0.71, 0.41–0.67, and 0.43–0.74 for models 1a, 1b, 2a, and 2b, respectively. The median AUC values for all models in the training cohorts were between 0.59–0.60. All models showed a wider range of AUC values in the validation cohorts. The AUC value distribution for all models in the training and validation cohorts are presented in the violin plots in Figure 3. The minimum and maximum sensitivity in the training cohorts were 18–64%, 31–71%, 0–65%, and 33–73% for models 1a, 1b, 2a, and 2b, respectively. The median sensitivity for all models in the training cohorts was between 42–58%. All models showed lower median sensitivity in the validation cohorts, except for model 2b. The minimum and maximum positive predictive value (PPV) in the training cohorts were 42–71%, 41–85%, 48–73%, and 43–86% for models 1a, 1b, 2a, and 2b, respectively. The median PPV for all models in the training cohorts was between 68–70%. All models showed a lower median PPV in the validation cohorts, except for model 2a. In all four models, the clinical tumor size was ranked as the most important clinical characteristic followed by age. The diagnostic performance parameters of the clinical models (100 iterations) are shown in Table 3.

### 2.5. RQS and TRIPOD

This study scored a radiomics quality score (RQS) of 58% (21 out of 36 points) (Table S2). The score of the transparent reporting of a multivariable prediction model for individual prognosis or diagnosis (TRIPOD) checklist was 67% (18 out of 27 applicable items) (Table S3).

## 3. Discussion

Accurate preoperative prediction of axillary lymph node metastasis can assist in clinical decision-making regarding the extent of axillary surgery and radiation therapy, and provide essential prognostic information. In this study, clinical models and radiomics models based on T2-weighted dedicated axillary MRI features with node-by-node analysis were investigated for the preoperative prediction of axillary lymph node metastasis. The different sets of features selected at each split resulted in a wide range of AUC values and did not allow for the development of a final radiomics prediction model. The performance of the clinical models (AUC values between 0.41–0.74) was lower compared to the radiomics models (AUC values between 0.48–0.89) in the training cohorts. The validation results of both models showed a wider range of diagnostic performance parameters compared to the training results possibly explained by the small dataset, the methodology used for selection and model building, and potential overfitting. The wide AUC range in the clinical models leads us to the hypothesis that the small dataset contains unseen biological covariates, and that therefore the wide AUC range in the radiomics models cannot be explained by variations in imaging alone.

To the best of our knowledge, this is the first study investigating the role of MRI-based radiomics for the prediction of axillary lymph node metastasis in breast cancer patients by extracting features from delineated axillary lymph nodes. Previously published articles investigated the same topic by extracting the features from the delineated breast tumor [15,20,21]. These articles showed promising validation results with AUC values between 0.77–0.82. In this recent study, initially, the small ROI volumes were seen as a reason for the inconclusive results. If an ROI contains a low number of voxels, it may not be possible to calculate meaningful radiomics features [22]. However, after the subanalysis excluding ROI volumes less than 50 voxels, the AUC values were between 0.53–0.82 and 0.41–0.83 for the training cohorts for models 1a and 2a, respectively, which highlights the effects of differences in scan acquisition and reconstruction parameters. Furthermore, the skewed data in this recent study may have caused inconsistent results compared to the previous studies as models tend to favor the more common outcome.

To date, only two previously published articles extracted features from delineated lymph nodes for radiomics and deep learning analyses. The first article used a neural network to develop prediction models in head and neck cancer [23]. The second article developed a radiomics model based on CT images of colorectal cancer patients [24]. Both studies showed that there is potential by delineating lymph nodes for radiomics and deep learning analysis for the classification of positive and negative lymph nodes. The differences in results compared to this recent study may be due to the variety of implementation of the different steps in the radiomics workflow and the chosen imaging modality (CT vs. MRI).

The diagnostic performance of dedicated axillary T2W MRI for axillary lymph node staging has previously been investigated using node-by-node analysis [12]. Schipper et al. showed AUC values between 0.78–0.88, with a good interobserver agreement (kappa = 0.70). The current analysis with MRI-based radiomics using dedicated axillary T2W MR images suggested that the quantitative analysis did not exceed the qualitative analysis by the radiologists. It was decided to only perform radiomics analyses using the T2W MR images, as previous research indicated that diffusion-weighted images and apparent diffusion coefficient measurements have no added value for the axillary lymph node staging [12,25]. Furthermore, a recently published article has shown that the evaluation of axillary lymph nodes with dedicated axillary MRI is comparable to standard breast MRI with a complete field of view of the axillary region [25]. However, the majority of the breast MRI examinations are still performed with an incomplete field of view of the axillary region [9]. In addition, the coronal view of the dedicated axillary MRI possibly provides more accurate delineations compared to the transversal view of the standard breast MRI, which could be of added value to the radiomics analysis.

Most radiomics studies suffer from small and heterogeneous datasets collected from different imaging systems. In this current study, a great advantage for the radiomics analyses was the prospectively collected set of MR images on the same MRI scanner using an equal acquisition protocol with the patients in corresponding positions. Despite the prospectively collected dataset, a number of acquisition and reconstruction parameters varied depending on the patient. Furthermore, the different sets of features selected in every training cohort resulted in a wide range of AUC values and did not allow the development of a final radiomics prediction model. This could be justified by two theories: (i) The variations in acquisition and reconstruction parameters significantly affected the value of radiomics features, resulting in non-comparable data points; or (ii) Radiomics features do not have an added value in the prediction of axillary lymph nodes metastasis. However, theory (ii) is less likely, as radiomics models performed well in some splits. Future MRI phantom and reproducibility studies should investigate the effect of MR image acquisition and reconstruction parameters on feature values to determine repeatable and reproducible features. We nevertheless believe that it is also important to publish inconclusive radiomics results since publication bias seems to play a role in this research field, with only 6% of the radiomics articles presenting negative results [26].

This study also has certain limitations. The large skewness of the data with only 7% positive axillary lymph nodes was a drawback for the analyses. The skewness of the data was addressed by splitting the dataset using two different strategies and by using repeated cross-validation in the training cohort. However, it is important to note that the ratio of node-positive (19%) and node-negative (81%) breast cancer patients in this study is comparable to the clinics. Besides the skewness of the data, the included number of patients was relatively low for radiomics analysis and selecting only node-positive patients in strategy 2 decreased the number even further. However, since the dedicated axillary MRI is not included in the breast MRI protocol and no similar public dataset is available, it is not possible to expand this current dataset. Lastly, manual delineation of the axillary lymph nodes was performed by one researcher, which potentially could be a major limitation of the findings because of the susceptibility of inter- and intra-observer variabilities [27]. Although this issue has been addressed in this current study by developing models based on only robust features for varying breast tumor delineations [28]. Based on the assumption that breast and lymph node delineations on MRI are comparable, varying delineations did not affect the radiomics results. However, this topic needs to be thoroughly investigated in future studies.

## 4. Materials and Methods

### 4.1. Patient Population

Consecutive women with histopathologically proven breast cancer, who had undergone dedicated axillary MRI between August 2012 and October 2014, followed by sentinel lymph node biopsy (SNLB) or axillary lymph node dissection (ALND), were considered for inclusion. Patients were excluded if they had undergone neoadjuvant systemic therapy before axillary surgery and in the case of ductal carcinoma in situ only. This study was approved by the local medical ethics committee, and the requirement of written informed consent was waived due to the retrospective study design. Fifty of the dedicated axillary T2W and diffusion-weighted MR images were earlier described by Schipper et al. for axillary lymph node staging, and 90 of the dedicated axillary T2W and gadofosveset-enhanced MR images were earlier described by Van Nijnatten et al. for axillary lymph node staging [12,29].

### 4.2. Clinical and Pathological Characteristics

Clinical and pathological data were derived from the patients’ medical records: age, clinical TNM stage, pathological TNM stage, tumor histology, tumor grade, breast cancer subtype, and type of axillary surgery. Lymph nodes with isolated tumor cells (≤0.2 mm) and micrometastases (>0.2–≤2.0 mm) were considered negative, while those with macrometastases (>2.0 mm) were considered positive.

### 4.3. MRI Acquisition

The dedicated axillary MR images were performed using a 32-channel cardiac coil on a 3.0 Tesla scanner (Achieva, Philips Healthcare, Best, the Netherlands). During the MRI examination, the patient was positioned in a supine position with the ipsilateral arm elevated. The anatomical confines of the dedicated axillary MR images were between the humeral head and the inferior border of the scapula. The MRI protocol included an unenhanced three-dimensional T2W turbo spin-echo sequence without fat suppression (pixel size, 1.25 × 1.25 mm; repetition time, 2000 ms; echo time between 150–202 ms; echo train length, 52 or 66; flip angle, 90°; acquisition slice thickness, 2.5 mm; reconstruction slice thickness, 1.25 mm), a contrast-enhanced T1-weighted sequence, and a diffusion-weighted imaging sequence with fat suppression.

### 4.4. MRI Lymph Node Delineation

**Figure 4:**
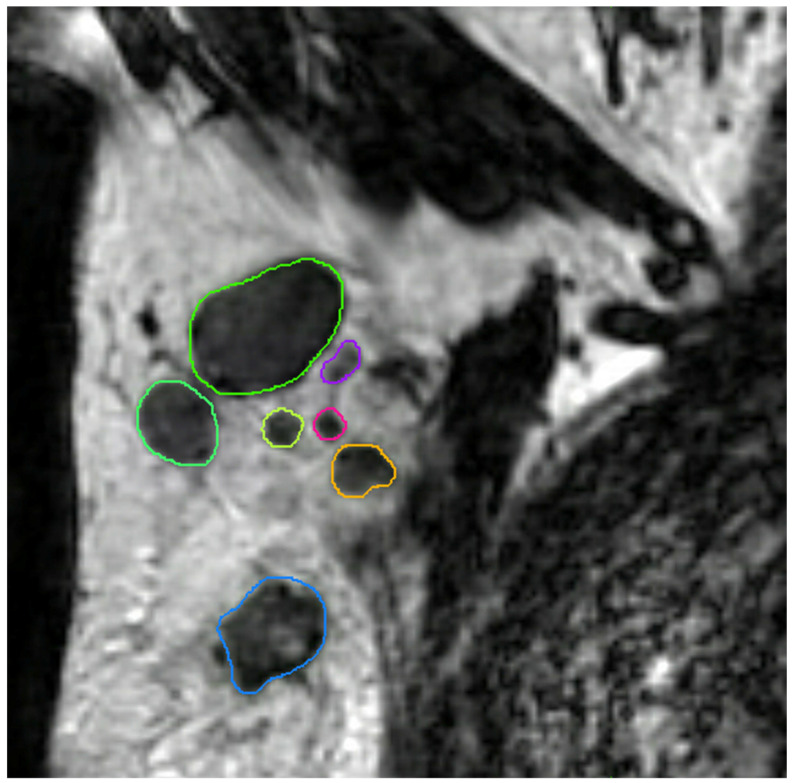
Coronal T2-weighted dedicated axillary MR image of a 55-year old woman with invasive breast cancer, who was treated with mastectomy and axillary lymph node dissection (pT1N2). The MR image demonstrates an example of delineations of lymph nodes in the right axilla on the MIM software.

All axillary lymph nodes of each dedicated axillary T2W MR image were manually delineated in three dimensions using MIM software (version 6.9.4, MIM Software Inc., Cleveland, OH, USA) by a medical researcher (S.S.) with three years of experience in axillary lymph node imaging validated by a dedicated breast radiologist (M.L.) with eleven years of experience (Figure 4). No clinical information and pathology results were available during delineation and validation. The delineated lymph nodes were subsequently matched with their histopathological findings (node-by-node matching). Reliable node-by-node matching was obtained using single-photon emission computed tomography-X-ray computed tomography (SPECT-CT) in patients undergoing SLNB, and an anatomical map was used for patients undergoing ALND. The exact procedure of the node-by-node matching was previously described by Schipper et al. [30].

### 4.5. MRI Preprocessing and Feature Extraction

Image preprocessing of the T2W images was performed after delineation. Bias field correction was applied to every T2W MR image using MIM software to correct for non-uniform grayscale intensities caused by field inhomogeneities. To ensure better comparability of voxel intensities, additional image normalization and discretization was performed by the open-source Pyradiomics software (version 2.2.0) prior to feature extraction [31]. For discretization, grayscale values were aggregated with a fixed bin width of 10, which ensured the recommended amount of bins between 30–130 [31]. Resampling was not required, as all images consisted of isotropic voxels of equal size 1.25 mm^3^. Quantitative radiomics features were extracted from the delineated regions using the Pyradiomics software. The extracted features can be subdivided into the following classes: first-order statistics, three-dimensional shape-based, gray level co-occurrence matrix, gray level run length matrix, gray level size zone matrix, neighboring gray-tone difference matrix, and gray level dependence matrix.

### 4.6. Radiomics Feature Selection and Model Development

**Figure 5:**
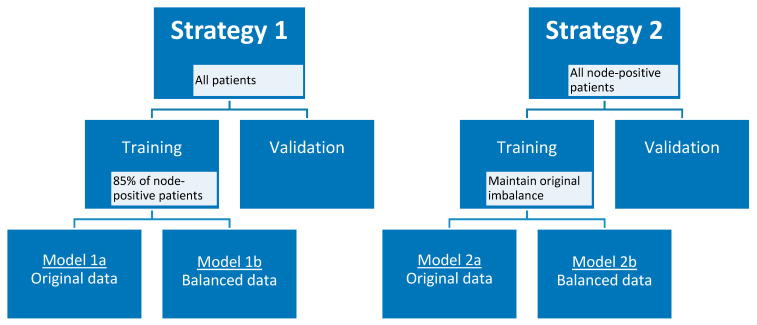
Model strategies.

Taking into account the small skewed dataset and the unavailability of an external validation dataset, the data were randomly divided into training and validation cohort 100 times using two different strategies to create a more balanced training cohort. In the first strategy, 85% (12 out of 14) of the node-positive (i.e., patients with axillary lymph node metastasis at final pathology) breast cancer patients were selected in the training cohort, and all remaining node-positive and node-negative (i.e., patients without axillary lymph node metastasis at final pathology) patients in the validation cohort, considering each axillary lymph node as an individual data point when training the model. In the second strategy, only the lymph nodes of patients with node-positive breast cancer were considered as individual data points when training and validating the model. To maintain the original class imbalance of the node-positive patients, 10 patients were selected in the training cohort. For both strategies, additional models were developed using a random undersampled balanced training cohort. All lymph nodes of one patient were always included in either the training cohort or the validation cohort, and therefore each split caused a varying number of positive lymph nodes in each cohort. Feature selection started with the removal of near-zero variance features followed by the removal of highly correlated features using the Pearson pairwise correlation greater than 0.95. Subsequently, recursive feature elimination with bagged trees was applied with repeated 5-fold cross-validation to select a maximum number of features in the training cohort. The number of features was chosen at the point when the addition of more features did not increase the diagnostic performance of the models. Random forest binary classification models were trained, using optimized random forest parameters (number of trees and features per node) for the training cohort, selecting the optimal number of features for each generated model. In addition, a separate set of models was generated using the same pipeline but by adding an additional feature selection step at the very beginning. In this step, features robust to the variability of manual delineations of breast tumors on MRI by four observers were selected according to three different cut-off values (intraclass correlation coefficient of >0.75, >0.80, and >0.90) [28]. Figure 5 provides an overview of strategies 1 and 2 with the different developed models.

### 4.7. Radiomics Subanalysis

A separate set of models was generated using the first and second strategies as described earlier on a dataset where ROIs with less than 50 voxels were excluded [31]. On these models, only the additional feature selection step with different intraclass correlation coefficient cut-off values was not performed.

### 4.8. Clinical Model Development

Clinical models were trained based on clinical characteristics available before the axillary surgery. Random forest models with bagged tree function for the prediction of axillary lymph node metastasis were trained and validated using the same strategies as described above, except for the feature selection step, which was only the removal of highly correlated clinical characteristics. These clinical models were used to indicate the effect of known and unknown patient’s biological covariates compared to a pure imaging-based model as well as to rank the importance of the clinical characteristics in this dataset using the Gini impurity method.

### 4.9. Statistical Analyses and Study Evaluation

The statistical analyses, including dataset splitting and balancing, feature selection, model development, and performance evaluation, were performed in R (version 3.6.3; http//www.r-project.org) using R studio (version 1.2.1335, Vienna, Austria) [32]. The performance of all models was assessed using the area under the receiver operating characteristics curve (AUC), sensitivity, specificity, positive predictive value (PPV), and negative predictive value (NPV). The Spearman correlation was used to calculate the correlation between the number of voxels per ROI and the corresponding pathological outcome. The radiomics workflow was evaluated using the radiomics quality score (RQS) [33]. This study followed the Transparent Reporting of a multivariable prediction model for Individual Prognosis Or Diagnosis (TRIPOD) guidelines [34].

## 5. Conclusions

In conclusion, based on our results dedicated axillary MRI-based radiomics with node-by-node analysis did not contribute to the prediction of axillary lymph node metastasis based on data where variations in acquisition and reconstruction parameters were not addressed. Larger datasets combined with MRI phantom data and reproducibility studies are necessary to determine if further radiomics research using dedicated axillary MR images for the prediction of axillary lymph node metastasis is of added value.

## Figures and Tables

**Table 1:** Patient, tumor, and treatment characteristics.

Characteristic	Value
No. of patients	75
Age (years) (median; IQR)	61 (51–68)
Clinical tumor size (mm) (median, IQR)	19 (13–28)
Clinical tumor stage (%)	
T1	41 (54.7)
T2	32 (42.7)
T3	2 (2.6)
Clinical nodal stage (%)	
N0	68 (90.7)
N1	7 (9.3)
Tumor histology (%)	
Invasive ductal	55 (73.3)
Invasive lobular	11 (14.7)
Mixed invasive ductal & lobular	3 (4.0)
Other	6 (8.0)
Tumor grade (%)	
1	17 (22.7)
2	42 (56.0)
3	16 (21.3)
Breast cancer subtype (%)	
ER + HER2−	55 (73.3)
ER + HER2+	6 (9.0)
ER − HER2+	2 (2.7)
Triple-negative	11 (14.7)
Not determined	1 (1.3)
Axillary surgery (%)	
SLNB	8 (10.7)
ALND	67 (89.3)

Abbreviations: ER, Estrogen receptor; HER2, Human epidermal growth factor receptor 2; IQR, interquartile range; SLNB, Sentinel lymph node biopsy; ALND, Axillary lymph node dissection.

**Table 2:** The diagnostic performance of the radiomics models (100 iterations) for the first and second strategy.

Diagnostic Parameters	Training				Validation				Training				Validation			
	Sens (%)	Spec (%)	PPV (%)	NPV (%)	Sens (%)	Spec (%)	PPV (%)	NPV (%)	Sens (%)	Spec (%)	PPV (%)	NPV (%)	Sens (%)	Spec (%)	PPV (%)	NPV (%)
**First Strategy**																
	**Model 1a**								**Model 1b**							
**Minimum**	30	71	46	62	0	78	0	98	53	50	55	72	0	57	0	98
**Median**	47	81	61	72	33	90	2	99	66	67	67	80	50	75	1	99
**Maximum**	66	91	78	79	100	97	22	100	83	85	83	88	100	88	10	100
**Second Strategy**																
	**Model 2a**								**Model 2b**							
**Minimum**	7	58	25	54	0	33	0	22	48	46	52	68	0	0	0	0
**Median**	50	81	62	74	33	76	50	71	66	68	67	80	64	60	50	75
**Maximum**	74	93	80	83	82	100	100	88	82	92	90	89	100	100	100	100

Abbreviations: NPV, negative predictive value; PPV, positive predictive value; sens, sensitivity; spec, specificity.

**Table 3:** The diagnostic performance of the clinical models (100 iterations) for the first and second strategy.

Diagnostic Parameters	Training				Validation				Training				Validation			
	Sens (%)	Spec (%)	PPV (%)	NPV (%)	Sens (%)	Spec (%)	PPV (%)	NPV (%)	Sens (%)	Spec (%)	PPV (%)	NPV (%)	Sens (%)	Spec (%)	PPV (%)	NPV (%)
**First Strategy**																
	**Model 1a**								**Model 1b**							
**Minimum**	18	64	42	65	0	40	0	99	31	46	41	42	0	14	0	97
**Median**	50	86	68	72	0	91	0	99	58	74	70	64	50	64	1	99
**Maximum**	64	93	71	78	100	99	18	100	71	92	85	73	100	88	9	100
**Second Strategy**																
	**Model 2a**								**Model 2b**							
**Minimum**	0	55	48	61	0	0	10	34	33	45	43	43	0	0	10	0
**Median**	42	85	68	72	39	80	69	73	57	75	70	63	61	53	43	67
**Maximum**	65	100	73	80	100	100	73	84	73	91	86	74	100	100	100	86

Abbreviations: NPV, negative predictive value; PPV, positive predictive value; sens, sensitivity; spec, specificity.

## Data Availability

The data presented in this study are available on reasonable request from the corresponding author. Due to privacy restrictions the data are not publicly available.

## Supplementary Materials

The following are available online at https://www.mdpi.com/2072-6694/13/4/757/s1, Supplementary Material A includes a list of how often each feature was chosen in the 100 iterations for each model (excel file). Supplementary Material B includes Figure S1: Violin plots for the radiomics models developed using the first (A) and second (B) strategy with additional feature selection step (ICC > 0.75): AUC value distributions (100 iterations) for the four models (1a, 1b, 2a, and 2b) in both the training and validation cohort, Figure S2: Violin plots for the radiomics models developed using the first (A) and second (B) strategy with additional feature selection step (ICC > 0.80): AUC value distributions (100 iterations) for the four models (1a, 1b, 2a, and 2b) in both the training and validation cohort, Figure S3: Violin plots for the radiomics models developed using the first (A) and second (B) strategy with additional feature selection step (ICC > 0.90): AUC value distributions (100 iterations) for the four models (1a, 1b, 2a, and 2b) in both the training and validation cohort, Figure S4: Violin plots for the radiomics models with the exclusion of ROIs < 50 voxels developed using the first strategy and second strategy: AUC value distribution (100 iterations) for the two models (1a and 2a) in both the training and validation cohort, Table S1: he diagnostic performance of the radiomics models (100 iterations) with the exclusion of ROIs < 50 voxels for the first and second strategy, Table S2: Radiomics Quality Score, Table S3: TRIPOD Checklist.
