# Supplementary material for: Dedicated Axillary MRI-Based Radiomics Analysis for the Prediction of Axillary Lymph Node Metastasis in Breast Cancer
Source: Cancers (Basel). 2021 Feb 12;13(4):757. doi: 10.3390/cancers13040757 (PMC7917661; doi:10.3390/cancers13040757)
Supplement: Supplementary file 1 [file cancers-13-00757-s001.zip › cancers-1059365 - supplementary/Table 2.docx]

**Table 2.** The diagnostic performance of the radiomics models (100 iterations) for the first and second strategy.

| **Diagnostic parameters** | **Training** | | | | **Validation** | | | | | **Training** | | | | **Validation** | | | |
| --- | --- | --- | --- | --- | --- | --- | --- | --- | --- | --- | --- | --- | --- | --- | --- | --- | --- |
|  | **Sens (%)** | **Spec**  **(%)** | **PPV**  **(%)** | **NPV**  **(%)** | **Sens**  **(%)** | **Spec**  **(%)** | **PPV**  **(%)** | **NPV**  **(%)** | **Sens**  **(%)** | | **Spec**  **(%)** | **PPV**  **(%)** | **NPV**  **(%)** | **Sens**  **(%)** | **Spec**  **(%)** | **PPV**  **(%)** | **NPV**  **(%)** |
|  | **First Strategy** | | | | | | | | | | | | | | | | |
|  | **Model 1a** | | | | | | | | | **Model 1b** | | | | | | | |
| **Minimum** | 30 | 71 | 46 | 62 | 0 | 78 | 0 | 98 | 53 | | 50 | 55 | 72 | 0 | 57 | 0 | 98 |
| **Median** | 47 | 81 | 61 | 72 | 33 | 90 | 2 | 99 | 66 | | 67 | 67 | 80 | 50 | 75 | 1 | 99 |
| **Maximum** | 66 | 91 | 78 | 79 | 100 | 97 | 22 | 100 | 83 | | 85 | 83 | 88 | 100 | 88 | 10 | 100 |
|  | **Second Strategy** | | | | | | | | | | | | | | | | |
|  | **Model 2a** | | | | | | | | | **Model 2b** | | | | | | | |
| **Minimum** | 7 | 58 | 25 | 54 | 0 | 33 | 0 | 22 | 48 | | 46 | 52 | 68 | 0 | 0 | 0 | 0 |
| **Median** | 50 | 81 | 62 | 74 | 33 | 76 | 50 | 71 | 66 | | 68 | 67 | 80 | 64 | 60 | 50 | 75 |
| **Maximum** | 74 | 93 | 80 | 83 | 82 | 100 | 100 | 88 | 82 | | 92 | 90 | 89 | 100 | 100 | 100 | 100 |

Abbreviations: NPV, negative predictive value; PPV, positive predictive value; sens, sensitivity; spec, specificity.
