# Supplementary figures and images for: Dedicated Axillary MRI-Based Radiomics Analysis for the Prediction of Axillary Lymph Node Metastasis in Breast Cancer
Source: Cancers (Basel). 2021 Feb 12;13(4):757. doi: 10.3390/cancers13040757 (PMC7917661; doi:10.3390/cancers13040757)

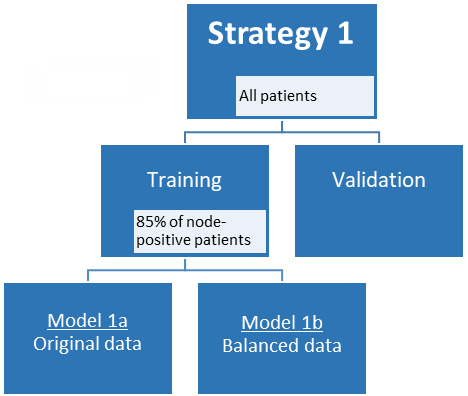

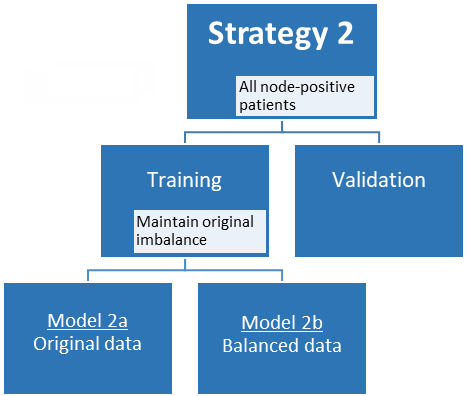


**Figure 5.** Model strategies

Supplement: Supplementary file 1 [file cancers-13-00757-s001.zip › cancers-1059365 - supplementary/Figure 5.docx]
